# A reassessment of Marquesan *Ochrosia* and *Rauvolfia* (Apocynaceae) with two new combinations

**DOI:** 10.3897/PhytoKeys.4.1599

**Published:** 2011-07-12

**Authors:** David H. Lorence, Jean-François Butaud

**Affiliations:** 1 National Tropical Botanical Garden, 3530 Papalina Road, Kalaheo, HI 96741, USA; 2 Consultant en foresterie et botanique polynésienne, P.O. Box 52832, 98716 Pirae, Tahiti, French Polynesia

**Keywords:** Apocynaceae, French Polynesia, Marquesas Islands, *Neisosperma*, *Ochrosia*, *Rauvolfia*

## Abstract

A reassessment of collections of Marquesan Apocynaceae assigned to the genera *Neisosperma* Raf.*, Ochrosia* Juss., and *Rauvolfia* L. revealed that two nomenclatural changes are necessary: 1) transfer of *Neisosperma brownii* Fosberg & Sachet to the genus *Ochrosia*, as *Ochrosia brownii* (Fosberg & Sachet) Lorence & Butaud, **comb. nov.**, and 2) transfer of *Ochrosia nukuhivensis* Fosberg & Sachet to *Rauvolfia* as *Rauvolfia nukuhivensis* (Fosberg & Sachet) Lorence & Butaud, **comb. nov**. As a result, two species each of *Ochrosia* and *Rauvolfia* are recognized from the Marquesas Islands, all endemic. Recent field work has yielded important new data on their distribution, habitat, and conservation status. It is recommended that all four species should be added the IUCN Red List at the Critically Endangered (CR) category.

## Introduction

In his Flora of Southeastern Polynesia, Forrest Brown (1935) incorrectly interpreted three Apocynaceae collections he made at 800 to 900 meters on Nuku Hiva and Fatu Hiva in the Marquesas Islands as belonging to the widespread coastal Indo-Pacific species, *Ochrosia parviflora* (G. Forst.) Henslow. The latter is now considered a synonym of *Ochrosia oppositifolia* (Lam.) K. Schum., or alternatively of *Neisosperma oppositifolium* (Lam.) Fosberg & Sachet(Nicolson and Fosberg 2004; Smith 1988), a species not known from the Marquesas but occurring in the Austral archipelago. Based on these three rather poor, incompletely flowering and fruiting collections, Fosberg and Sachet (1972) described two species in *Ochrosia* Juss., one each from Nuku Hiva and Fatu Hiva, and one in *Neisosperma* Raf. from Nuku Hiva. Fosberg(1981) later described a species of *Rauvolfia* L. based on a Sachet collection from Hiva Oa.

During the preparation of an account of Apocynaceae for the Vascular Flora of the Marquesas Islands and Flore de la Polynésie française, additional field work by J-FB and study of additional herbarium specimens have greatly increased our knowledge of the Marquesan taxa and their correct identity. For example, collections from field work associated with the project resulted in the discovery of a new species of *Lepinia*, a genus previously not even known from the Marquesas (Lorence and Wagner 1997). Critical study of the type collections of the four previously described species of Marquesan Apocynaceae, all apparently endemic, coupled with new collections and field observations has resulted in a reassessment of their generic status.

### *Ochrosia* or *Neisosperma* ?

Traditionally *Neisosperma* [incorrectly spelled *Neiosperma* by Fosberg & Sachet (1972)] was separated from *Ochrosia* based on two morphological features: the gynoecium possesses a nectary comprised of two thin lobes or scales present in the former but lacking in the latter; secondly, the fruit endocarp is fibrous and muricate with no air cavity in the former and fibrous but relatively smooth-surfaced with two air cavities in the latter (Smith 1988: 44). Disintegration of the surrounding pericarp and mesocarp by weathering, however, reveals the endocarp of *Ochrosia* to be fibrous as well (Lorence and Butaud, pers. obs. 2010). In both cases the fruit consists of usually two mericarps.

Based on morphological and molecular evidence, recent classifications of Apocynaceae tend to recognize only a single genus *Ochrosia* (Endress et al. 2007; Simões et al. 2007; Mabberley 2008). Molecular studies of 19 species of *Ochrosia* using cpDNA (the rps 16 intron) and ITS sequences revealed that *Ochrosia* species formed a strongly supported clade sister to and nested within a paraphyletic *Neisosperma* clade (Hendrian and Kondo 2007a, b). However, additional data from combined rps16 intron plus ITS sequences showed a more robust clade of *Ochrosia* sensu lato, supported by 100% bootstrap value, supporting the inclusion of *Neisosperma* into *Ochrosia* and recognition of only one genus (Hendrian and Kondo 2007c), a conclusion we adopt here. Nevertheless, it is possible that additional studies may show that the genus *Neisosperma* could be resurrected in a narrower concept potentially including two species from Marquesas and one species from the Austral Islands.

## Methodology

All measurements given herein are taken from dried herbarium specimens, although certain features such as shapes were supplemented with information from field notes, alcohol-preserved specimens, and digital photos. Measurements are presented in the descriptions as follows: length × width (or length × width × thickness in the case of fruits and seeds), followed by units of measurement (mm or cm). All specimens cited in this paper have been seen by the authors. Specimens from the following herbaria were studied: AD, BISH, K, MO, MU, PAP, PTBG, and US. The area of occupancy (distribution) for these species was calculated using herbarium collection data and field observations, and the conservation status is proposed following the IUCN Red List Category criteria (IUCN^ 2001^; www.iucnredlist.org/info/categories_criteria2001).

## The Marquesan species of *Ochrosia*

### Key to species of *Ochrosia* in the Marquesas Islands

**Table:** 

1a	Calyx lobes c. 2 mm long; corolla lobes 6-7 mm long; mericarps ellipsoid 37-53 × 27-37 mm; Nuku Hiva	*Ochrosia brownii*
1b	Calyx lobes c. 2.5 mm long; corolla lobes 9-10 mm long; mericarps ovoid-ellipsoid 61-78 × 32-44 mm; Fatu Hiva	*Ochrosia fatuhivensis*

### Ochrosia brownii

(Fosberg & Sachet) Lorence & Butaud comb. nov.

urn:lsid:ipni.org:names:77112734-1

http://species-id.net/wiki/Ochrosia_brownii

Figs 1A, B

Neisosperma brownii Fosberg & Sachet, Micronesica 8: 49, 1972 [as *Neiosperma*]). [Basionym]

#### Type.

**Marquesas Islands:** Nuku Hiva: without precise locality, 15 July 1921, F. B. H. Brown 541 (Holotype BISH-500905!).

*Ochrosia parviflora* sensu F. Br., non (G. Forst.) Henslow

#### Description.

*Tree* to 13 m tall, trunk to 24 cm diam., branchlets glabrous, leafy twigs 4−4.5 mm in diam, terete, drying wrinkled, older leafless twigs 6−7 mm in diam., latex white. *Leaves* opposite on smaller branchlets, ternate on larger branchlets, petiolate, leaf axils with dark brown, linear-digitate colleters 1−1.5 mm long secreting pale yellow resin; blades obovate-elliptic, 9.4−16.5 × 3.2−6.4 cm, base narrowly cuneate, attenuate, apex shortly acuminate, glabrous, discolorous, drying brown above, yellowish-brown below, when fresh green to yellow green above, pale green below, both surfaces glossy, secondary veins 15−20 on each side, secondary and tertiary veins visible above, prominulous beneath, margins conspicuously and tightly revolute; petioles 17−33 mm long, 1.7−2 mm in diam. *Inflorescence* terminal, 7−12 cm long, trichotomous, branching to the third degree, with 2 primary branches at apex of peduncle, each with 12−18 flowers, glabrous, axes with small, scale-like bracts; flowers on pedicels 1.5−4 mm long, 5-merous; calyx lobes obtuse to rounded, 2 × 2.5 mm; corolla in bud to 10 mm long; corolla at anthesis white, fragrant, corolla tube 6−7 × 3 mm, corolla lobes 5, contorted to the right, 6−7 × 2−25 mm, rounded at apex; ovary conical, 0.7−0.8 mm long, bicarpellate, style 0.8 mm long, stigmatic head ovoid, 0.7-0.8 mm long with tuft of hairs at apex and green ring (but no collar) at base, nectary 0.3 mm, 2-lobed, the lobes alternating with and partly covering the carpels. *Infructescence* with peduncle c. 17 cm long; fruits composed of two fleshy mericarps, when fresh orange at maturity, ellipsoid, 37−53 × 27−37 × 25−36 mm, mesocarp orange, 5 mm thick; endocarp externally fibrous, internally woody, 31−48 × 23−33 × 19−27 mm, the fibers to 1.5 mm in diam. *Seed* 2 per fruit, ellipsoid, 25 × 23−26 × c. 4 mm.

#### Distribution.

Nuku Hiva, Marquesas Islands, originally known only from the type collected in the vicinity of the Toovii plateau at about 900 m (Brown 1935). The species was rediscovered by Jean-François Butaud in 2003 on Nuku Hiva where a single tree and some juveniles were located on the Vaioa plateau (at Matahamo, also called Vaipupui) SE of Toovii.

#### Ecology.

This species occurs in evergreen wet forest between 730 and 900 m elevation, with species of *Hibiscus*, *Ixora*, *Metrosideros*, *Pandanus*, *Phyllanthus*, *Premna*, *Wikstroemia*, *Xylosma*, and ferns including *Asplenium australasicum* (J. Sm.) Hook., *Histiopteris incisa* (Thunb.) J. Sm., and *Microsorum grossum* (Langsd. & Fisch.) Brownlie.

#### Conservation status.

When evaluated using the IUCN criteria for endangerment (IUCN^ 2001^) *Ochrosia brownii* falls into the Critically Endangered (CR) category, which designates species facing the highest risk of extinction in the wild. IUCN Red List Category: **Critically Endangered** (CR) B1a, b; B2a, B2b (i–iii): B1, extent of occurrence estimated to be less than 100 km2, and B1a, known to exist at only a single location; B1b (i-iii), continuing projected decline in (i) extent of occurrence, (ii) area of occupancy, and (iii) area, extent and quality of habitat; B2, area of occupancy estimated to be less than 10 km2, and B2a, a single population known. B2b (i–iii), habitat continuing decline inferred. The suitable habitat for *Ochrosia brownii* on Nuku Hiva (*c.* 340 km2) is indicated as an endangered environment, threatened by human activity (deforestation and fire), feral animals, and invasive plants, reducing the extent of the forest.

#### Specimens examined.

**Marquesas Islands:** Nuku Hiva: Matahamo, Vaioa, fin de la piste forestière, 758 m, 2 Jan. 2003, Butaud 8 (PAP); Matahamo, Vaioa, 758 m, 9 Feb. 2003, Butaud 10 (PAP); Vaipupui, amont de la piste forestière menant à la dernière parcelle de pins, 737 m, 27 Jan. 2010, Butaud & Benne 2586 (PAP).

#### Discusison.

Since 2006, a conservation plan proposed by the Environment Direction of French Polynesia has been implemented, with conservation *ex situ* (seed collection and conservatory plantings) and *in situ* (fencing enclosure of the population and enrichment planting of seedlings inside).

**Figure 1.**
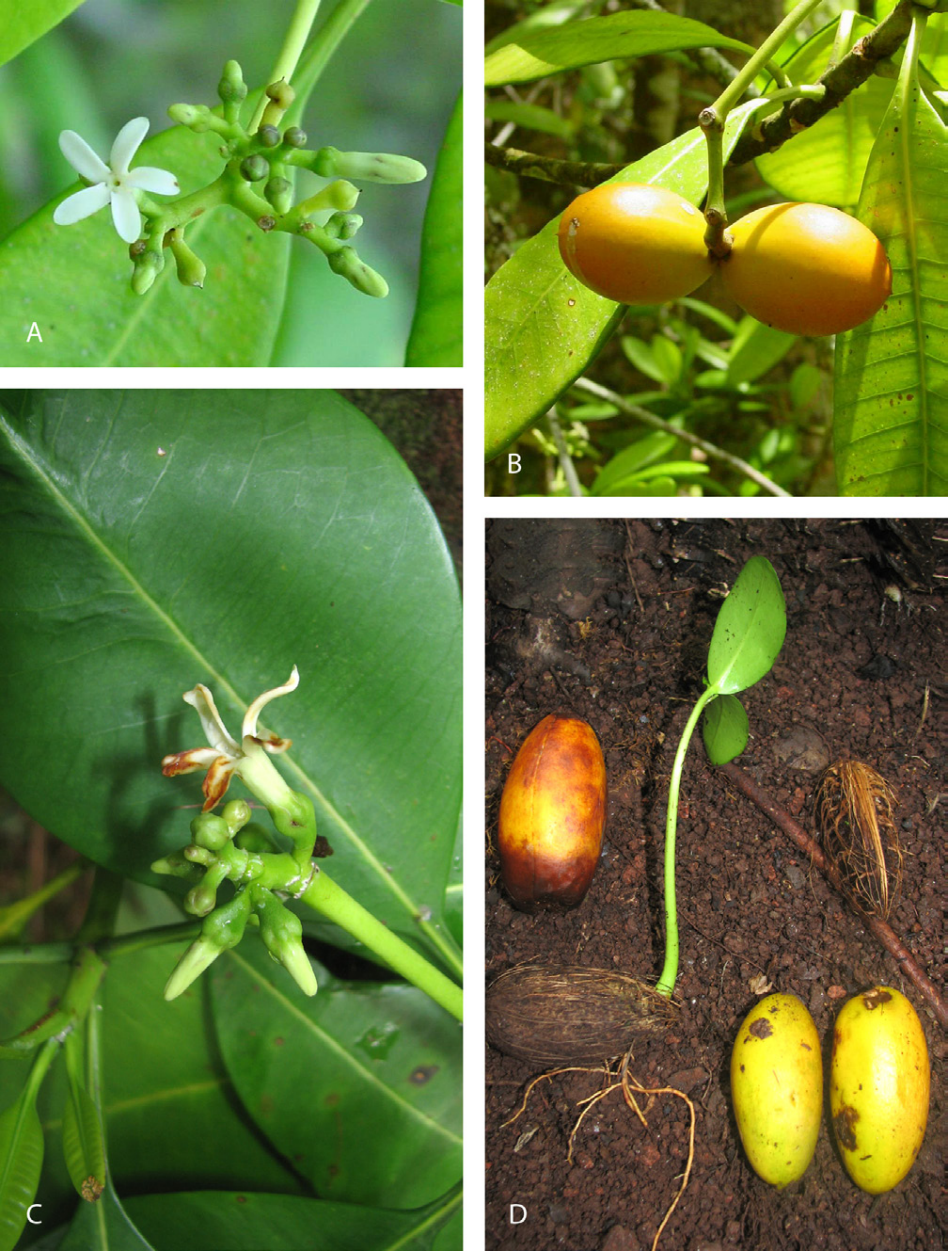
**A, B** *Ochrosia brownii*. **A** inflorescence and open flower and buds showing right-contorted corolla lobes (Vaipupui, Nuku Hiva, Feb. 2003, Butaud unvouchered) **B** branchlet with apocarpous fruit composed of paired mericarps (Vaipupui, Nuku Hiva, Butaud 8); **C, D** *Ochrosia fatuhivensis*. **C** inflorescence with open flower and buds, Hanativa (Fatu Hiva, Nov. 2009, Butaud 2458) **D** ripe mericarps, seed showing fibers and germinating seed (Hanativa, Fatu Hiva, Nov. 2009, Butaud 2458).

### Ochrosia fatuhivensis

Fosberg & Sachet, Micronesica 8: 48 (1972)

http://species-id.net/wiki/Ochrosia_fatuhivensis

Figs 1C, D 

#### Type.

**Marquesas Islands**: Fatu Hiva: Oia [Ouia], c. 800 m, 19 Jan. 1922, F. B. H. Brown 886 (Holotype BISH-500706!).

#### Description.

*Tree* 10−14 m tall, trunk 15−20 cm in diam., branchlets glabrous, leafy twigs 2.5−7 mm in diam., bark wrinkled when dry, latex white. *Leaves* opposite on smaller branchlets, apparently ternate on larger branchlets; blades elliptic, 5.8−20.2 × 2.4−10.1 cm, base narrowly cuneate, apex shortly acuminate, glabrous, discolorous, when fresh dark green above, pale green below, drying brown, margins slightly revolute; secondary veins 9−20 on each side, prominulous on both surfaces, intersecondaries and tertiaries prominulous on both surfaces; petioles 14−36 mm long. *Inflorescence* terminal, tri- or quadrichotomous, branching twice, cymose-corymbiform, 52−88 mm long, axes and flowers glabrous, sessile, with 3 primary branches (6−)20−64 × c. 2 mm, subquadrangular, bracts ovate-triangular, 1−1.2 × 1−1.2 mm, ultimate axes with flowers crowded in cymules; flowers with pedicels 2−5 mm long, each with a single bract, calyx cup 2.5 mm long, calyx lobes 5, triangular-ovate, 2.5 × 3 mm, apex obtuse; corollas in bud to 10 mm long; corolla at anthesis white, fragrant, corolla tube 7−8 × 3 mm, corolla lobes 5, contorted to the right, 9−10 × 2−2.5 mm; ovary and nectary not seen. *Infructescence* with peduncle c. 11.5 cm long. *Fruits* apocarpous, composed of 2 fleshy mericarps, when fresh orange at maturity, ovoid-ellipsoid, 61−78 × 32−44 × 30−40 mm, mesocarp c. 7 mm thick; endocarp 54−72 × 27−40 × 23−34 mm, fibrous without, woody within, single-seeded. *Seed* 2 per fruit, ellipsoid, 29−37 × 18−19 × 3−4 mm.

#### Distribution.

Marquesas Islands, Fatu Hiva where originally known only from the type collected in Ouia Valley at 800 m.

#### Ecology.

This species was recently rediscovered in 2009 by Jean-François Butaud and Ravahere Taputuarai following the directions of Joseph Mititai. This population in Hanativa valley, a remote hanging valley north of Ouia, consists of 13 adult plants and numerous juveniles and seedlings at 480 m elevation. It occurs in mesic to wet forest with species of *Aleurites*, *Allophylus*, *Artocarpus*, *Boehmeria*, *Cerbera*, *Inocarpus*, *Macropiper*, *Metrosideros*, *Musa troglodytarum* L.*, Pandanus, Phyllanthus, Pipturus argenteus* (G. Forst.) Wedd., *Wikstroemia*, the introduced *Coffea arabica* L. and *Dioscorea* spp.,and the ferns *Nephrolepis biserrata* (Sw.) Schott*, Pteris comans* G. Forst., and *Tectaria jardinii* (Mett. ex Kuhn) E. D. Br.

#### Conservation status.

*Ochrosia fatuhivensis* is currently known only from a single population. When evaluated using the IUCN criteria for endangerment it falls into the Critically Endangered (CR) category, which designates species facing the highest risk of extinction in the wild. IUCN Red List Category: **Critically Endangered** (CR) B1a, b; B2a, B2b (i–iii): B1, extent of occurrence estimated to be less than 100 km2, and B1a, known to exist at only a single location; B1b (i-iii), continuing projected decline in (i) extent of occurrence, (ii) area of occupancy and (iii), area, extent and quality of habitat; B2, area of occupancy estimated to be less than 10 km2, and B2a, a single population known. B2b (i–iii), habitat continuing decline inferred. The suitable habitat for *Ochrosia fatuhivensis* on Fatu Hiva (*c.* 85 km2) is indicated as an endangered environment, threatened by human activity (deforestation and fire), feral animals, and invasive plants, reducing the extent of the forest.

#### Specimen examined.

**Marquesas Islands:** Fatu Hiva: Hanativa, Affluent Sud de la vallée, vallon perché, 481 m, 7 Novembre 2009, Butaud, Taputuarai & Mititai 2458 (PAP).

#### Discussion.	

Fosberg & Sachet (1972) suggested *Ochrosia fatuhivensis* may be related to *Ochrosia compta* K. Schum. of Hawaii, but no molecular-phylogenetic studies have been carried out involving these two species. The local name is ho’ei in Fatu Hiva, similar to the name holei used for species of *Ochrosia* in Hawaii (Wagner et al. 1990). On Fatu Hiva the seeds were eaten in time of famine according to Brown (1935) and several present day inhabitants.

## The Marquesan species of *Rauvolfia*

A single species of *Rauvolfia*, *Rauvolfia sachetiae* Fosberg was described from Hiva Oa, Marquesas based on a single collection (Fosberg 1981). Recent collections and field observations of *Rauvolfia* from Nuku Hiva were thought to represent this species. However, a re-examination of the holotype of *Ochrosia nukuhivensis* Fosberg & Sachet (*Brown 432*, BISH) reveals that it possesses distinctive whorled leaves and dark, linear or digitate, resiniferous colleters in the leaf axils and adjacent petiole characteristic of most Pacific *Rauvolfia* species, although the type lacks fruits. In *Ochrosia* the colleters are generally shortly conical or scale-like and usually obscured by a copious resinous exudate. Additional morphological differences separating these two genera include in *Rauvolfia* the corolla lobes are left-contorted (sinistrorse), presence of a well-developed annular nectary disc, and the presence of a conspicuous membranous basal collar on the style-head, whereas in *Ochrosia* corolla lobes are right-contorted (dextrorse), the nectary is absent or consists of 2 small, poorly developed thin lobes alternating with the carpels, and the style head sometimes has a ring of longer hairs near its base but no collar (M. Endress, pers. comm. 2010).

A recent Nuku Hiva collection, *Perlman 10014* distributed under the name *Rauvolfia sachetiae*, compares very well with *Brown 432*, the type of *Ochrosia nukuhivensis*, and clearly represents the same species. Furthermore, *Perlman 10014* has subglobose or globose, syncarpous fruits characteristic of many Pacific species of *Rauvolfia* in which normally each of the two carpels contains two ovules, but frequently one aborts so that at maturity the fruit is usually 2-seeded, but sometimes one carpel does not fully develop, resulting in a 1-seeded syncarpous drupe. In *Ochrosia* the fruits consist of a pair of well separated, distinct or bally basally connate oblong mericarps (Wagner et al. 1990). This Nuku Hiva species differs in leaf and calyx morphology from *Rauvolfia sachetiae* on Hivaoa, and the two species may be separated by the following key.

### Key to species of *Rauvolfia* in the Marquesas Islands

**Table:** 

1a	Leaves narrowly ovate to narrowly elliptic or oblong, L:W ratio 2.9:1 to 3:1, secondary veins 21-25 on each side; calyx total width distally 3.2-4 mm; calyx lobes 1.5-2 × 1.5-2 mm	*Rauvolfia nukuhivensis*
1b	Leaves ovate to elliptic, L:W ratio 1.7:1 to 2:1, secondary veins 18-19(-21) on each side; calyx total width distally, 2-2.5 mm; calyx lobes 1-1.2 × 1-1.2 mm	*Rauvolfia sachetiae*

### Rauvolfia nukuhivensis

(Fosberg & Sachet) Lorence & Butaud comb. nov.

urn:lsid:ipni.org:names:77112735-1

http://species-id.net/wiki/Rauvolfia_nukuhivensis

Figs 2A, B, C

Ochrosia nukuhivensis Fosberg & Sachet, Micronesica 8: 48, 1972. [Basionym]

#### Type.

**Marquesas Islands:** Nuku Hiva: without precise locality, ca. 1000 m, 20 June 1921, F. B. H. Brown 432(Holotype BISH-500695!).

#### Description.

*Tree* 8−15 m tall, trunk 40−50 cm in diam., bark furrowed, pale orange to beige in color, leafy branchlets 3−3.5 mm in diam., glabrous, bark wrinkled, brown, leafless branchlets 6 mm in diam., gray-brown, latex white. *Leaves* ternate, leaf axils and adjacent petiole bases with brown, glandular digitate colleters 1−2 mm long; blades narrowly ovate to narrowly elliptic or oblong-elliptic, 6−17.6 × 1.5−6.2 cm, base narrowly cuneate to cuneate, apex acute, chartaceous, glabrous, when fresh pale green, discolorous, margin slightly revolute, secondary veins 21−25 on each side, intersecondary and tertiary veins prominulous on both surfaces, petioles 12−36 × 1.2−1.5 mm, adaxially flattened. *Inflorescences* terminal, displaced by terminal shoot growth, 5−7 × 7−10 cm, including the verticellate peduncles 1.7−5.4 × 2 mm, branchlets slightly zigzag, glabrous, branching three to four times, ultimate branches trichotomous, bracteoles 1.5 mm long, triangular, acute; calyx total width distally 3.2−4 mm, calyx lobes 5, broadly triangular-ovate, 1.5-2 ×1.5-2 mm, obtuse at apex; corolla cream color when fresh, tube 8−13 mm long, lobes 2−3.5 × 2−2.5 mm, slightly contorted to the left, anthers 1.2-1.3 mm long, narrowly triangular-subulate, sagittate at base, ovary conical, 1.5 mm long, nectary disc annular, 0.4-0.5 mm long, style 5 mm long, stigma head cylindric, 0.4 mm long, with membranous collar at base. *Fruit* subglobose when mature, 12−15 mm in diam., fleshy, turning black at maturity, drupaceous with 1−3 seeds. *Seeds* compressed, obliquely oblong-ovoid, with one straight and one curved edge, base sub-truncate, apex diagonal from straight edge upward to a blunt point, sides coarsely and shallowly rugose.

#### Distribution.

Marquesas Islands, known only from Nuku Hiva between 198 and 627 m elevation.

#### Ecology.

*Rauvolfia nukuhivensis*is very rare and localized with fewer than 50 living trees currently known in valley or plateau dry forest with species of *Cerbera*, *Ficus*, *Maytenus*, *Phyllanthus*, *Sapindus*, and *Xylosma*. Currently known in the Terre-Deserte region in the valleys of Motuee, Hakaavao, Haahopu, Haatuatua, Hakaoa, Tapueahu and Uea, and on the plateaus of Tohuahee, Vaiteheii, Maauu, and Putata. As many dead trees as living trees are known. Formerly known from the north coast of Nuku Hiva, between the Haataivea peninsula and behind Aakapa village.

#### Conservation status.

The suitable habitat for *Rauvolfia nukuhivensis* on Nuku Hiva (*c.* 340 km2) is indicated as an endangered environment, threatened by human activity (deforestation, exploitation for leaves and bark), feral animals, and invasive plants, reducing the extent of the forest. Following the criteria and categories of IUCN (2001) IUCN Red List Category: **Critically Endangered** (CR) B1a, B1b (i-iii), D; B1, extent of occurrence estimated to be less than 100 km2, and B1a, severely fragmented (several small subpopulations); B1b (i-iii), continuing projected decline in (i) extent of occurrence, (ii) area of occupancy, and (iii) area, extent, and quality of habitat; D population size estimated to number fewer than 50 mature individuals (fewer than 50 total individuals).

#### Specimens examined.

**Marquesas Islands:** Nuku Hiva: Tapueahu (labels erroneously says Matatekouaehi) Valley, 650 ft. (198 m), 1 July 1988, Perlman 10014 (AD, BISH, K, MO, MU, P, PAP, PTBG, US); Terre Déserte, branche gauche de la moyenne vallée de la Baie Marquisienne, face au Keiaki, 340 m, 30 July 1987, Florence 8434 (BISH, PAP, US); broad interfluve above Uea Valley, near Baie Marquisienne, 20 Apr. 1964, Decker 2046 (US); Ivipuovoteahi, NO de Keiaki, 400 m, 8°55’ S, 140°12’ W, February 1987, Jourdan 128 (PAP); Terre Deserte, Pipiheihe, 400 m, 8 April 1989, Toutain 4275 (PAP); Plateau de Maauu, au Sud du deuxième vallon de la forêt naturelle, 456 m, 9 April 2004, Butaud 418 (PAP).

#### Discussion.

Collectors’ notes indicate the leaves and bark are used for traditional medicine, another factor likely contributing to the decline of this species. Since 2006, a conservation plan lead by the Environment Direction of French Polynesia was implemented, with conservation *ex situ* (seed collection and conservation plantings) and *in situ* (enclosure of several populations). Local name: tueiao

**Figure 2.**
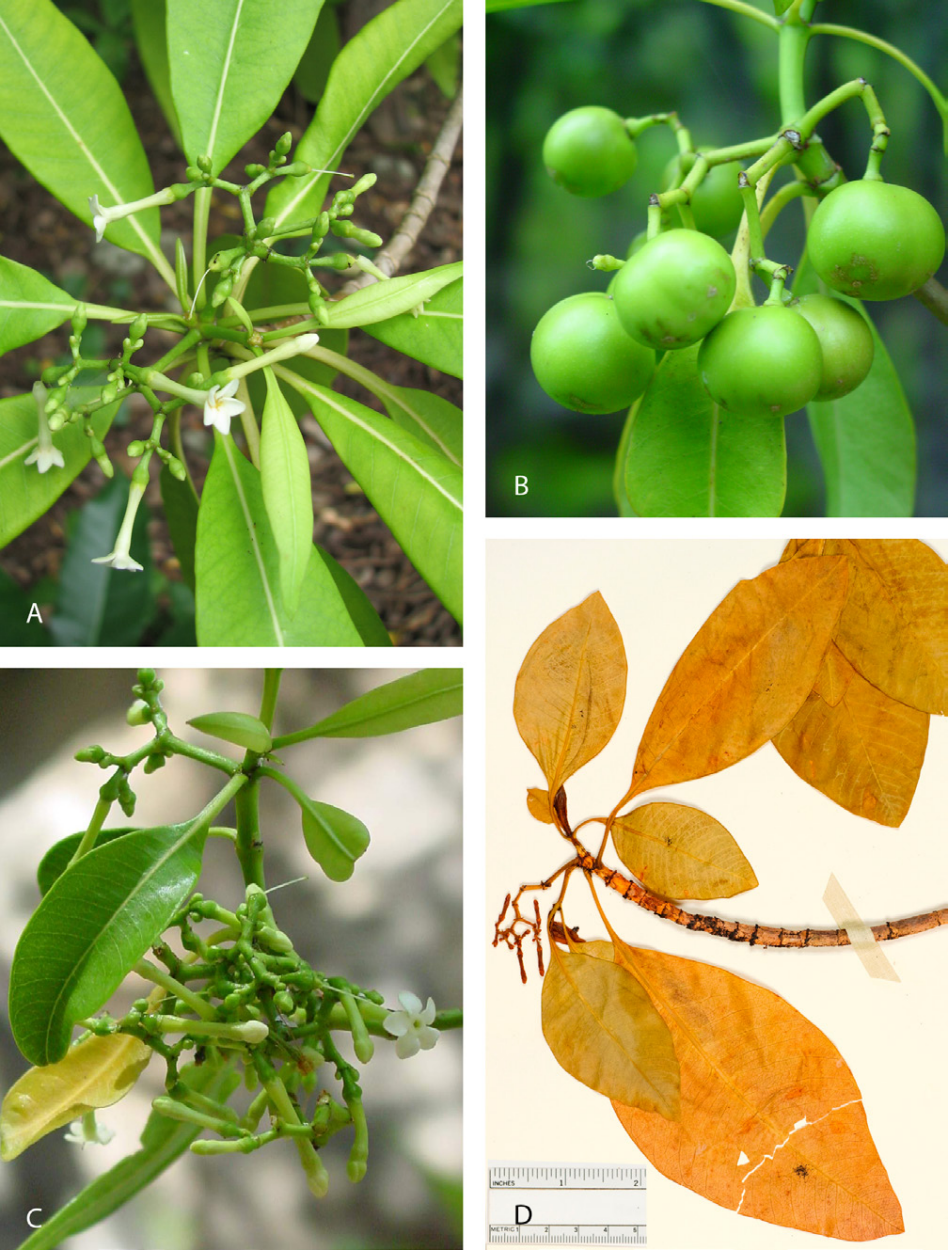
**A, B, C,** *Rauvolfia nukuhivensis* **A** branchlet with leaves and inflorescence with open flowers, and buds with left-contort corolla lobes (Terre-Deserte, Nuku Hiva, May 2007, Butaud (unvouchered) **B** infructescence with syncarpous green fruits (Maauu, Nuku Hiva, April 2004, Butaud 418) **C** inflorescence with buds and open flower (Keiaki, Nuku Hiva, 27 Dec. 2002, J.-F. Butaud unvouchered); **D** *Rauvolfia sachetiae*, portion of isotype specimen showing leaves and inflorescence (Hiva Oa, above Taaoa, 15 Jan. 1975, Sachet 2115, Isotype BISH-578658).

### Rauvolfia sachetiae

Fosberg, Smithsonian Contr. Bot. 47: 21, 1981.

http://species-id.net/wiki/Rauvolfia_sachetiae

Fig. 2D 

#### Type:

**Marquesas Islands**: Hiva Oa: dry crest above Taaoa, SW of village, 350 m, M.-H. Sachet 2115 (Holotype US!; Isotype BISH-598409!).

#### Description.

*Tree* 6−10 m tall, trunk 20−25 cm in diam., bark furrowed, branches more or less horizontal, twigs and leaves in whorls of 3−4, entire plant glabrous, latex abundant; branchlets 4−6 mm in diam., proximal internodes to 9 cm long, distal ones 0.5−1 cm long toward end of season’s growth, terminal buds with abundant clear, brown resin. *Leaves* elliptic to broadly elliptic, 5.5−22 × 2.5−6.8 cm apex acute to obtuse, base acute to occasionally obtuse, slightly decurrent on petiole, glabrous, chartaceous, light to bright yellowish green when dried, secondary veins 12−15 on a side, brochidodromous, forming a wide angle with costa, secondary and tertiary veins prominulous on both surfaces; petioles 15−35 × 1−2 mm in diam. with 20−30 brown digitate, glandular colleters 0.5−1.2 mm long in each leaf axil. *Inflorescences* terminal, pseudoaxillary by displacement, compound cymes 3−6 × 8−12.5 cm including the corollas, peduncles verticellate, 1.5−3 cm long, 3−6 times ramified, branches open, widely divergent, ultimate branches trichotomous with central branch usually represented by a single pedicellate flower, most flowers early caducous from all but the most distal branches, each lateral branch subtended by a scale-like, ovate-triangular bract 1−1.5 × 1−1.5 mm, a bract pair subtending each lateral bud, axils of bracts glandular as those of leaves, pedicel of central flower 2−2.5 mm long, lateral buds of a triad subsessile, globose, tending to develop into a new triad with only the central flower of a triad developing to anthesis; calyx turbinate-campanulate, lobed almost to base, lobes 1.5−2 × 1.5−1.7 mm, imbricate, obtuse, margins thin; corolla yellow in bud, at anthesis cream colored, 12−14 mm long including limb, tube 9.5−14 × 1−1.5 mm in diam. medially, slightly dilated at apex and base, lobes slightly left-contorted, ovate, 2.5−3.5 × 2 mm, obtuse or rounded at apex, anthers narrowly ovate, somewhat bluntly acuminate, sagittate at base, subsessile, 1.5 mm long, inserted 1.5 mm below throat, a very few hairs around and below insertion on inside surface of corolla tube; ovary surrounded by cup-like nectary disk with minutely crenulate margin, carpels oblong, free distally, united in lower half, style glabrous, 7−9 mm long, style head thickened, cylindrical (not seen: floral description from Fosberg 1981). *Fruit* subglobose to globose, 12−15 mm long, fleshy, turning black at maturity (at least when dry), apex rounded or emarginate, subtended by persistent spreading calyx. *Seed* 1 by abortion (only one mature fruit examined) 12 × 8 × 5 mm, obliquely oblong-ovoid, with one straight and one curved edge, base sub-truncate, apex diagonal from straight edge upward to a blunt point, sides coarsely and shallowly rugose.

#### Distribution.

Marquesas Islands, Hiva Oa, previously known only from a single tree at the type locality above Taaoa. In 2011 one dead trunk probably representing this species was found in a small gulch above Tanaeka valley, at around 430 m elevation (Butaud, pers. obs.).

#### Ecology.

Dry secondary forest or shrubland with *Casuarina*.

#### Conservation status.

Despite careful searches this species has not been found since the last collection was made at the type locality in 1977. In 2010 the area was observed to be overgrazed by goats and invaded by *Syzygium cumini*.(L.) Skeels (Butaud, pers. obs.). *Rauvolfia sachetiae*falls into the **Critically Endangered (CR)** category, which designates species facing the highest risk of extinction in the wild. IUCN Red List Category: **Critically Endangered** (CR) B1a, b; B2a, B2b (i–iii): B1, extent of occurrence estimated to be less than 100 km2, and B1a, known to exist at only a single location; B1b (i-iii), continuing projected decline in (i) extent of occurrence, (ii) area of occupancy, and (iii) area, extent and quality of habitat; B2, area of occupancy estimated to be less than 10 km2, and B2a, a single population known. B2b (i–iii), continuing habitat decline inferred. The suitable habitat for *Rauvolfia sachetiae* on Hiva Oa (*c.* 315 km2) is indicated as an endangered environment, threatened by human activity (deforestation and fire), feral animals, and invasive plants, reducing the extent of the forest.

#### Specimens examined.

**Marquesas Islands:** Hiva Oa: Ridge above Taaoa, SW of village, 350 m, 20 Nov 1974, Sachet & Decker 1885 (BISH, CBG, CHR, L, MO, NSW, P, PTBG, US), Taaoa, sur la presqu’île, versant SW, 350 m, 8 March 1977, Schäfer & Oliver 5293 (BISH, CBG, CHR, MO, NSW, PTBG, US).

## Supplementary Material

XML Treatment for Ochrosia brownii

XML Treatment for Ochrosia fatuhivensis

XML Treatment for Rauvolfia nukuhivensis

XML Treatment for Rauvolfia sachetiae
